# Effects of sunitinib on endothelial dysfunction, metabolic changes, and cardiovascular risk indices in renal cell carcinoma

**DOI:** 10.1002/cam4.2910

**Published:** 2020-04-09

**Authors:** Silvia Lai, Maria Ida Amabile, Sandro Mazzaferro, Anna Paola Mitterhofer, Angelo Mazzarella, Alessandro Galani, Giovanni Imbimbo, Rosario Cianci, Marzia Pasquali, Alessio Molfino, Study Group on Geriatric Nephrology of the Italian Society of Nephrology (SIN)

**Affiliations:** ^1^ Department of Translational and Precision Medicine Sapienza University of Rome Rome Italy; ^2^ Department of Cardiovascular, Respiratory, Nephrological, Anesthesiology and Geriatric Sciences Sapienza University of Rome Rome Italy; ^3^ Department of Clinical and Experimental Sciences University of Brescia Brescia Italy; ^4^ Nephrology and Dialysis Unit Policlinico Umberto I Rome Italy

**Keywords:** cardiovascular risk, endothelial dysfunction, hypertension, metabolism, renal cell carcinoma, renal function, sunitinib

## Abstract

**Background:**

Sunitinib is a standard treatment for metastatic renal cell carcinoma (RCC). Currently, the data available on the effects of sunitinib on endothelial dysfunction, metabolic changes, and cardiovascular (CV) risk factors are limited, and we aimed to evaluate these aspects in patients with RCC after a short period of treatment.

**Methods:**

Patients affected by metastatic RCC were enrolled and evaluated before starting sunitinib (T0) and after 40 days of treatment (T1) by the flow‐mediated dilation (FMD), carotid intima media thickness (IMT), ankle‐brachial pressure index (ABI), and 24‐hour proteinuria. We also assessed serum metabolic and nutritional parameters at T0 and T1.

**Results:**

Twenty patients (7 female), with a mean age of 61.4 ± 12.0 years, were studied. Overtime, we observed a reduction in estimated glomerular filtration rate (*P* = .002), FMD (*P* = .001) and in fasting plasma glucose levels (*P* = .04), as well as an increase in plasma insulin (*P* < .001), HOMA‐IR (*P* < .01), and serum total cholesterol levels (*P* = .01). Moreover at T1 we found a significant increase in systolic and diastolic blood pressure (*P* ≤ .001) and 24‐hour proteinuria (*P* < .001) compared to baseline, whereas no changes in IMT and ABI were detected.

**Conclusion:**

The changes observed overtime after sunitinib treatment in terms of markers of early endothelial dysfunction, blood pressure, as well as in glucose/insulin metabolism and proteinuria may contribute to increase CV risk in RCC patients and suggest a strict follow‐up in this setting. Larger evidences are mandatory to confirm our observations.

## Graphical abstract

Our study aimed to evaluate endothelial dysfunction markers, metabolic changes and cardiovascular risk indices in patients with metastatic renal cell carcinoma treated with sunitinib. The results obtained appears clinically interesting, considering that the negative effects we observed were obtained after a short period of time (40 days) from sunitinib administration. Our data suggest to oncologists to perform a strict follow‐up in this clinical setting.

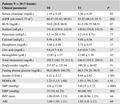

## 1 INTRODUCTION

Renal cell carcinoma (RCC) is the most common type of renal cancer and represents 2%‐3% of all adult malignancies.^1^ Targeted therapies with small molecule tyrosine kinase inhibitors (TKIs), regulate some important pathways, including cell proliferation and apoptosis, and are currently the first line treatments for many cancers.^2^ The TKIs seem to have less toxicity compared to conventional cytotoxic chemotherapeutic agents, with a relatively high therapeutic window, even if TKIs have important and common side effects, including fatigue, hypertension, hand‐foot syndrome, rash, gastrointestinal toxicities with anorexia, nausea, diarrhea, and myelosuppression, that are reversible after discontinuation of the treatment.^3^, ^4^ However, concerns exist on therapies’ long‐term side effects, such as endocrine‐related adverse effects, including thyroid function, bone metabolism, adrenal function, and glucose metabolism beyond to endothelial dysfunction, renal and cardiovascular damage.^3^, ^4^ In particular, heart failure, reduced left ventricular ejection fraction and myocardial infarction were observed in cancer patients undergoing selected targeted agents.^5^ The TKIs include molecules inhibiting the vascular endothelial growth factor (VEGF), such as sunitinib which represents nowadays an important novel therapy for RCC.^2^ Few data are available on the potential negative effects of sunitinib, and, although studies showed an association between sunitinib dose intensity and better outcomes, the management of its side effects is not well defined.^6^ In this light, research is focusing on amelioration of patients tolerability with TKIs, including sunitinib, to improve the outcomes.

The aim of the present study was to evaluate endothelial dysfunction markers, metabolic changes, and cardiovascular risk indices in patients with metastatic RCC after a short period of treatment with sunitinib.

## 2 PATIENTS AND METHODS

The study protocol was approved by our Local Clinical Research Ethics (Azienda Policlinico Umberto I) and it conforms to the principles outlined in the Declaration of Helsinki and later amendments. We obtained written consent from each patient before the enrolment. We performed an observational prospective study on clinically stable patients affected by metastatic RCC treated with sunitinib, at the Divisions of Nephrology and Oncology, Policlinico Umberto I, Sapienza University of Rome, Italy. Taking into account that some studies showed the development of the most common side effects after a median of 4 weeks from sunitinib initiation in patients with RCC,^7^ clinical, laboratory, and instrumental parameters in our cohort were evaluated at baseline (T0) and after 40 days (T1) from the initiation of sunitinib.

All the patients received sunitinib, orally, at recommended dose of 50 mg/d for 4 weeks each 6 weeks. In case of grade 2 or 3 toxicity according to National Cancer Institute‐Common Toxicity Criteria, therapy was planned to be continued at a reduced dose (37.5 or even 25 mg/d) or suspended.

### 2.1 Inclusion criteria

The inclusion criteria included age >18 years and <80 years, WHO performance status 0‐2 and life expectancy ≥12 weeks, patients with metastatic RCC in whom the treatment of choice was sunitinib and patients signing the informed consent form.

### 2.2 Exclusion criteria

The exclusion criteria included the use of corticosteroids, the presence of heart failure, severe cardiovascular disease, liver failure or chronic liver disease, chronic kidney disease 4/5 stage KDOQI (eGFR < 30 mL/min), unstable or uncompensated respiratory disease. We also excluded pregnant or breastfeeding patients, and those with missing clinical data.

### 2.3 Laboratory measurements

Blood was collected in the morning after an overnight fasting (≥12 h). The estimated glomerular filtration rate (eGFR) was calculated with the abbreviated modification of diet in renal disease formula (MDRD).^8^ In all the patients, the levels of fasting plasma glucose (mg/dL), hemoglobin (Hb) (g/dL), total serum cholesterol (mg/dL), triglycerides (mg/dL), electrolytes, serum uric acid (mg/dL) were measured using standard automated techniques. We assessed proteinuria (mg/d) by 24‐hour urine collection.

### 2.4 Blood pressure (BP) measurements

The BP measurements were carried according to the British Hypertension Society guidelines,^9^ using a standard automatic sphygmomanometer. Hypertension was defined according to the international guidelines, as described previously.^10^ We have also determined ankle‐brachial pressure index (ABI) (normal values 0.9‐1).

### 2.5 Common carotid intima‐media thickness assessment (IMT)

Intima media thickness was calculated using a machine Toshiba Aplio xV (Toshiba Aplio xV, Toshiba American Medical Systems, Inc) with a 5‐ to 12‐MHz linear transducer, as described previously.^11^ The mean IMT was computed as the average IMT on both sides. Normal IMT values were 0.55‐0.9 mm.^11^

### 2.6 Flow‐mediated dilation brachial artery (FMD)

The FMD was assessed using the same Toshiba Aplio xV following a standardized protocol according to the Celermajer method.^12^, ^13^ Flow‐mediated vasodilation was expressed as: (diameter post‐hyperemia‐basal diameter/basal diameter) × 100, and it was considered normal if greater than 10%.

### 2.7 Statistical analyses

Shapiro‐Wilk test (normality test) was used to determine if the data set was well‐modeled by a normal distribution. All continuous variables were expressed as mean ± standard deviation and categorical variables were expressed as numbers (percentage). Not‐normally distributed variables were described using median (25th, 75th percentiles). One‐way ANOVA or Wilcoxon signed‐tank test were performed to determine differences between groups, as appropriate. Binomial test or chi‐squared test was used for the comparison of categorical data. All comparisons were two‐sided and a *P* < .05 was considered significant. Data management and analysis were performed using IBM^®^ SPSS^®^ Statistics 17 for Windows^®^ software (IBM Corporation, New Orchard Road Armonk).

## 3 RESULTS

### 3.1 Patient characteristics at baseline

Patient characteristics at baseline are indicated in Table 1. In summary, we enrolled 20 patients (7 female) with a mean age 61.4 ± 12.0 years, receiving sunitinib orally at 50 mg/d for 4 weeks every 6 weeks. All the participants showed median eGFR values (based on MDRD) (mL/min/1.73 m^2^) of 60.47 (53.42; 69.01) (Table 1), body weight (kg) of 65.2 ± 8.4 and a BMI (kg/m^2^) of 24.05 ± 2.32. Five patients (25% of our cohort) were affected by arterial hypertension and only one patient (5%) by chronic heart failure (NYHA class 1).

**Table 1:** Patient characteristics at baseline (T0) and after 40 d from sunitinib administration (T1)

Patients N = 20 (7 female) Clinical parameter	T0	T1	*P*
Serum creatinine (mg/dL)	1.19 ± 0.29	1.36 ± 0.29	.82
eGFR (mL/min/1.73 m^2^)	60.47 (53.42; 69.01)	53.52 (48.14; 55.7)	.002
BUN (mg/dL)	54.0 (26.0; 68.0)	61.5 (56.75; 68.5)	.07
Sodium (mEq/L)	141.0 (139.0; 142.0)	140.0 (139.0; 142.5)	.96
Potassium (mEq/L)	4.5 (4.20; 4.76)	4.2 (4.0; 4.71)	.73
Calcium (mEq/L)	9.49 ± 0.58	9.60 ± 0.56	.67
Phosphorus (mg/dL)	3.48 ± 0.58	3.75 ± 0.57	.17
Uric acid (mg/dL)	5.6 (4.7; 6.8)	6.6 (6.0; 7.25)	.17
Hemoglobin (g/dL)	12.83 ± 1.57	12.40 ± 1.76	.43
Total cholesterol (mg/dL)	192.5 (161.75; 211.5)	246.0 (192.5; 259.5)	.01
Triglycerides (mg/dL)	215.87 ± 122.44	180.76 ± 66.02	.51
Fasting plasma glucose (mg/dL)	96.0 (88.0; 100.75)	88.5 (79.25; 99.5)	.04
Insulin (UI/mL)	6.31 ± 2.12	9.69 ± 2.82	<.001
HOMA‐IR	1.23 (1.13; 1.68)	1.93 (1.59; 2.34)	<.01
SBP (mmHg)	124 ± 12.94	149.25 ± 9.77	<.0001
DBP (mmHg)	75 (70; 81.25)	90 (90; 95)	.001
IMT (mm)	1.00 (0.95; 1.13)	1.00 (0.96; 1.23)	.66
ABI	1.08 (1.03; 1.11)	1.02 (1.0; 1.11)	.68

Note

Data are shown as mean ± standard deviation or median (25th, 75th percentile).

Abbreviations: ABI, ankle brachial index; BUN, blood urea nitrogen; DBP diastolic blood pressure; eGFR, estimated glomerular filtration rate; HOMA‐IR, Homeostasis model assessment‐insulin resistance; IMT, intima media thickness; SBP, systolic blood pressure.

### 3.2 Clinical changes overtime

During the study period, eight patients (40%) reduced sunitinib dosage from 50 mg/d to 37.5 mg/d due to the development of side effects including mucositis, hand‐and‐foot syndrome, anemia and diarrhea.

After 40 days (T1) from the initiating dose of sunitinib, we observed a significant reduction, compared to baseline, in eGFR (*P* = .002), and fasting plasma glucose levels (Table1) (*P* = .04); in parallel, we found a significant increase in plasma insulin levels (*P* < .001) (Table 1) and in HOMA‐IR (*P* < .01) (Table 1).

**Figure 1:**
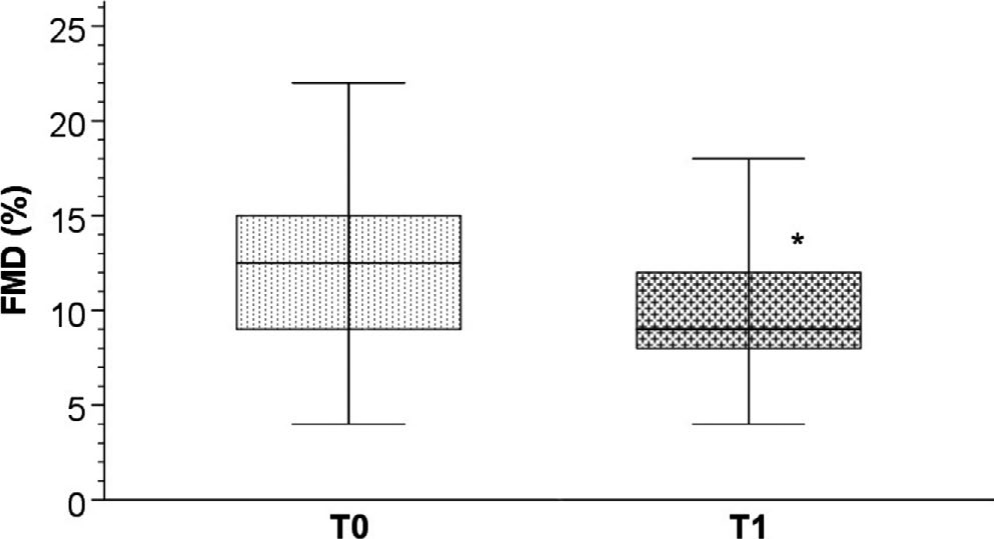
Bar charts with error bars. The median FMD (%) value was significantly higher at T0 with respect to T1 (12.5 (9.25; 15) vs 9 (8; 11.5); * *P* = .001). Boxes represent means; error bars indicate ± 1 SD. Abbreviations: FMD, flow mediated dilation; SD, standard deviation; T0, baseline; T1 after 40 days of sunitinib treatment

Also, at T1 we found significant lower median FMD (%) values with respect to baseline (12.5 (9.25; 15) vs 9 (8; 11.5); *P* = .001) (Figure 1).

**Figure 2:**
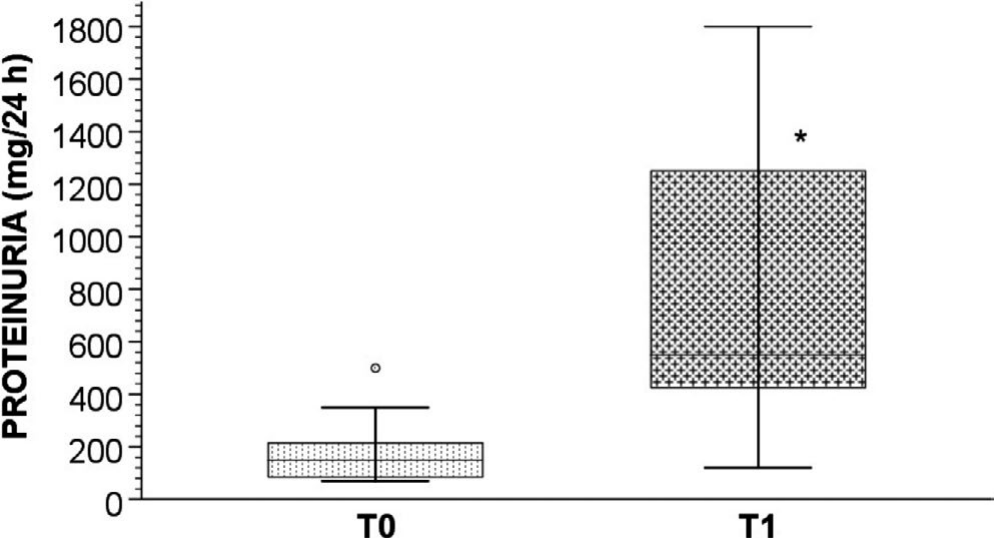
Bar charts with error bars. The median value of proteinuria (mg/24 h) was significantly lower at T0 with respect to T1 (150 (85; 215) vs 540 (425; 1250); *P < .001). Boxes represent means; error bars indicate ± 1 SD. Abbreviations: T0, baseline; T1, after 40 days of sunitinib treatment

Moreover we found a significant increase compared to baseline in systolic blood pressure (SBP) (*P* < .0001) (Table 1), diastolic blood pressure (DBP) (*P* = .001) (Table 1) and in median 24 hour‐proteinuria (mg) values (150 (85; 215) vs 540 (425; 1250); *P* < .001) (Figure 2). We also observed a significant increase in serum total cholesterol levels (*P* = .01) (Table 1), while we did not report a significant change in IMT and ABI, as well as in serum triglycerides (Table 1).

## 4 DISCUSSION

Sunitinib is a standard treatment for metastatic RCC.^2^ In particular, TKIs have largely improved the prognosis of patients with metastatic RCC, with apparently lower short‐term toxicity but with longer‐term side effects, including endocrine‐related adverse effects, endothelial dysfunction, renal and cardiovascular damage.^3^ Sunitinib targets primarily VEGF receptors (VEGF‐Rs), and platelet‐derived growth factor receptors (PDGF‐Rs). PDGF‐Rs are primarily expressed in fibroblasts and vascular mural cells and are involved in cell survival, proliferation, and migration.^14^ VEGF‐Rs are expressed in endothelial cells and are involved in vasculogenesis and angiogenesis.^14^, ^15^ Therefore, sunitinib acts also on the endothelial and vascular mural cells, with angiogenesis inhibition, but common adverse effects of this inhibition are hypertension, renal injury and cardiac dysfunction. Moreover, angiogenesis inhibitors increase endothelial activation that can lead to vascular dysfunction and accelerated atherosclerosis.^16^

In this study, we observed an increase in SBP, DBP, and proteinuria, representing indicators of renal damage and endothelial dysfunction. In addition, we observed a significant worsening of renal function with a reduction of FMD, which is an early marker of endothelial dysfunction.^13^

Güneş et al^17^ suggested to evaluate proteinuria and FMD for early detection of renal damage and endothelial disfunction. Another study^18^ reported that the activation of the endothelin‐1 and the renin suppression are involved in sunitinib‐induced hypertension, proteinuria, and renal damage, determining lower activity of the NO system; this effect, as reflected by the decrease in urinary nitric oxide (NO) metabolites and cyclic guanosine 3′,5′‐monophosphate, has been also described by Henri et al.^16^

Endothelial dysfunction determined by anti VEGF seems to be primarily responsible for vascular, renal and cardiac damage,^2^, ^19^ and it seems to act early. In fact, the results obtained in our cohort showed, after sunitinib treatment, a significant reduction of FMD (%), although it was not accompanied by changes in IMT and ABI, considered as systemic markers of atherosclerosis, likely due to the early stage of disease or therapy.

Thijs et al^20^ showed a reduction of endothelium vasodilation by reducing endothelial release of NO in animals one week after sunitinib administration, but the authors showed that lower endothelium‐dependent FMD did not precede the development of hypertension during sunitinib administration.

Lankhorst et al^18^ reported some possible mechanisms for sunitinib‐induced hypertension, as deregulation of NO, oxidative stress, activation of endothelin‐1, salt sensitivity and microvascular alteration and suggested that the cardiovascular effects of sunitinib are not only mediated by hypertension but likely by direct effects on the heart and endothelium.^18^ Sourdon et al^21^ showed that sunitinib may impair cardiovascular function by inducing a switch of cardiac metabolism to anaerobic glycolysis with reduced glucose uptake. In fact, some TKIs, including imatinib, sunitinib, pazopanib, or nilotinib, were associated with changes in glucose metabolism inducing hypoglycemia or hyperglycemia, although the molecular mechanism(s) are not completely clarified.^22^

Our results indicate, after sunitinib administration, a significant reduction in fasting plasma glucose levels in non‐diabetic patients, although remaining within the normal range and without documenting hypoglycemic and/or symptomatic events. Our observation is in line with the those reporting a significantly decreased plasma glucose concentrations, only in patients with diabetes during sunitinib administration.^23^, ^24^ In addition, we observed at T1 higher insulin plasma levels (and HOMA‐IR) that may be associated with the lower glucose levels detected.

Some case reports documented an improvement in glycemic control in diabetic patients during TKIs treatment or a significant decrease in plasma glucose levels in diabetic and nondiabetic patients after sunitinib treatment that resulted almost always reversible after drug discontinuation.^24^, ^25^ Hyperglycemia was described during therapy in 15% of patients with metastatic RCC.^23^

Agostino et al^25^ suggested that sunitinib may lower insulin clearance in subjects with metastatic RCC; indeed, sunitinib may reduce the kinase activity of the insulin receptor and therefore block insulin internalization and degradation. Some authors suggested that c‐KIT tyrosine kinase may alter blood glucose levels, considering that c‐KIT has been reported to be associated in experimental studies with pancreatic beta‐cell survival.^26^, ^27^ Other authors indicated the vascular damage in pancreatic islets as the potential responsible of the altered glycemic profile due to direct effect of TKIs on pancreatic cell mass.^28^ Instead, Billemont et al^29^ and Hagerkvist et al^30^ reported a possible impact of sunitinib on insulin resistance by interfering with insulin‐like growth factor‐1 in humans and by altering the hepatic glucose production in an animal model.

Louvet et al^31^ showed in mouse models that inhibition of platelet‐derived growth factor downstream‐mediated inflammatory response may counteract beta‐cell death and insulin resistance. Moreover, TKIs showed limited or no effect on lipids.^28^ In our study, we did not find a significant difference in serum triglycerides, although we described a significant increase in serum total cholesterol.

Moreover recent studies reported additional side effects with TKIs, including changes in thyroid, bone, and gonadal functions, fetal development, and adrenal function.^3^, ^22^

Our study has several limitations including the small number of RCC patients. We included patients treated with antiplatelet, antihypertensive therapies and with lipid‐lowering molecules potentially affecting the results here obtained. Additional data are necessary to clarify the underlying mechanisms responsible for renal, cardiovascular and metabolic negative effects in cause‐effect studies.

In conclusion, the modifications observed overtime after sunitinib treatment in terms of markers of early endothelial dysfunction, blood pressure, as well as in glucose/insulin metabolism and proteinuria might increase the cardiovascular risk in RCC patients treated with sunitinib. Larger studies are mandatory to confirm our results. However, a strict metabolic and cardiovascular follow‐up in this setting is recommended especially in order to manage these targeted therapies safely.

## CONFLICT OF INTEREST

The authors declare no conflict of interest.

## AUTHOR CONTRIBUTIONS

SL designed research, conducted research, analyzed data, and wrote the paper. MIA conducted research, analyzed data, and wrote the paper. SM conducted research, and reviewed the paper. APM reviewed the paper. An.Ma. collected the data. AG analyzed data and performed statistical analyses. GI collected the data. RC collected the data. MP collected the data. AM designed research, analyzed data, and wrote the paper.
